# Supplementary material for: Time-series transcriptome analysis identified differentially expressed genes in broiler chicken infected with mixed Eimeria species
Source: Front Genet. 2022 Aug 8;13:886781. doi: 10.3389/fgene.2022.886781 (PMC9393255; doi:10.3389/fgene.2022.886781)
Supplement: Supplementary file 2 [file DataSheet1.ZIP › 7dpi_KEGG.Gsea.1625115961950/KEGG_INOSITOL_PHOSPHATE_METABOLISM.html]

Details for gene set KEGG\_INOSITOL\_PHOSPHATE\_METABOLISM[GSEA]

|  || Dataset | TMM\_7dpi\_gct\_format\_7dpi\_gct\_format.Class\_4dpi.cls #PC\_versus\_NC.Class\_4dpi.cls #PC\_versus\_NC\_repos |
| Phenotype | Class\_4dpi.cls#PC\_versus\_NC\_repos |
| Upregulated in class | 0 |
| GeneSet | KEGG\_INOSITOL\_PHOSPHATE\_METABOLISM |
| Enrichment Score (ES) | -0.49438393 |
| Normalized Enrichment Score (NES) | -1.628198 |
| Nominal p-value | 0.0031256976 |
| FDR q-value | 0.07878966 |
| FWER p-Value | 0.5614 |
Table: GSEA Results Summary

  

Fig 1: Enrichment plot: KEGG\_INOSITOL\_PHOSPHATE\_METABOLISM      
 Profile of the Running ES Score & Positions of GeneSet Members on the Rank Ordered List

  

| SYMBOL | TITLE | RANK IN GENE LIST | RANK METRIC SCORE | RUNNING ES | CORE ENRICHMENT || 1 | PLCD4 | na | 176 | 1.084 | 0.0229 | No |
| 2 | PIK3C2B | na | 743 | 0.616 | -0.0030 | No |
| 3 | OCRL | na | 1555 | 0.366 | -0.0580 | No |
| 4 | PLCB4 | na | 2151 | 0.245 | -0.0993 | No |
| 5 | PLCD3 | na | 2185 | 0.238 | -0.0938 | No |
| 6 | INPP1 | na | 2880 | 0.141 | -0.1469 | No |
| 7 | PLCE1 | na | 2983 | 0.129 | -0.1509 | No |
| 8 | PLCD1 | na | 3024 | 0.123 | -0.1500 | No |
| 9 | PIP5K1C | na | 3125 | 0.111 | -0.1545 | No |
| 10 | PIP5K1B | na | 3713 | 0.039 | -0.2022 | No |
| 11 | TPI1 | na | 3900 | 0.016 | -0.2172 | No |
| 12 | INPP5J | na | 5608 | -0.160 | -0.3544 | No |
| 13 | PI4KB | na | 5680 | -0.167 | -0.3545 | No |
| 14 | PIP5K1A | na | 5711 | -0.171 | -0.3511 | No |
| 15 | PIP4K2B | na | 5735 | -0.173 | -0.3470 | No |
| 16 | IMPA1 | na | 5761 | -0.177 | -0.3430 | No |
| 17 | PLCG1 | na | 6241 | -0.228 | -0.3751 | No |
| 18 | INPP5A | na | 6714 | -0.276 | -0.4050 | No |
| 19 | PLCB1 | na | 7317 | -0.343 | -0.4434 | No |
| 20 | INPP5E | na | 7472 | -0.358 | -0.4438 | No |
| 21 | INPP5K | na | 8078 | -0.432 | -0.4794 | Yes |
| 22 | SYNJ2 | na | 8110 | -0.436 | -0.4668 | Yes |
| 23 | ALDH6A1 | na | 8228 | -0.450 | -0.4610 | Yes |
| 24 | ISYNA1 | na | 8240 | -0.451 | -0.4462 | Yes |
| 25 | ITPKA | na | 8252 | -0.453 | -0.4314 | Yes |
| 26 | PIK3CG | na | 8478 | -0.481 | -0.4335 | Yes |
| 27 | PTEN | na | 8620 | -0.503 | -0.4279 | Yes |
| 28 | IMPA2 | na | 9082 | -0.562 | -0.4469 | Yes |
| 29 | IPPK | na | 9226 | -0.587 | -0.4385 | Yes |
| 30 | INPPL1 | na | 9459 | -0.624 | -0.4362 | Yes |
| 31 | INPP5B | na | 9519 | -0.634 | -0.4191 | Yes |
| 32 | PIP4K2A | na | 9723 | -0.664 | -0.4130 | Yes |
| 33 | PI4KA | na | 9785 | -0.671 | -0.3947 | Yes |
| 34 | PIK3C2G | na | 10128 | -0.737 | -0.3977 | Yes |
| 35 | IPMK | na | 10143 | -0.741 | -0.3732 | Yes |
| 36 | PIK3CB | na | 10335 | -0.777 | -0.3621 | Yes |
| 37 | PLCG2 | na | 10697 | -0.853 | -0.3627 | Yes |
| 38 | PIK3CD | na | 10707 | -0.855 | -0.3337 | Yes |
| 39 | PLCB2 | na | 10772 | -0.872 | -0.3088 | Yes |
| 40 | PIK3CA | na | 10920 | -0.912 | -0.2894 | Yes |
| 41 | PIK3C3 | na | 11107 | -0.966 | -0.2714 | Yes |
| 42 | INPP4A | na | 11216 | -1.005 | -0.2455 | Yes |
| 43 | PIK3C2A | na | 11243 | -1.015 | -0.2124 | Yes |
| 44 | SYNJ1 | na | 11396 | -1.079 | -0.1876 | Yes |
| 45 | MINPP1 | na | 11522 | -1.126 | -0.1589 | Yes |
| 46 | PIKFYVE | na | 11699 | -1.238 | -0.1306 | Yes |
| 47 | INPP4B | na | 11768 | -1.304 | -0.0910 | Yes |
| 48 | ITPK1 | na | 11837 | -1.390 | -0.0483 | Yes |
| 49 | MIOX | na | 11974 | -1.805 | 0.0030 | Yes |
Table: GSEA details [plain text format]

  

Fig 2: KEGG\_INOSITOL\_PHOSPHATE\_METABOLISM      
 Blue-Pink O' Gram in the Space of the Analyzed GeneSet

  

Fig 3: KEGG\_INOSITOL\_PHOSPHATE\_METABOLISM: Random ES distribution      
 Gene set null distribution of ES for **KEGG\_INOSITOL\_PHOSPHATE\_METABOLISM**
